# Lost and changed meaning in life of people with Long Covid: a qualitative study

**DOI:** 10.1080/17482631.2023.2289668

**Published:** 2023-12-06

**Authors:** Marishelle Lieberwerth, Alistair Niemeijer

**Affiliations:** a University of Humanistic Studies, Utrecht, Netherlands; b Department of Ethics of Care, University of Humanistic Studies, Utrecht, Netherlands

**Keywords:** Meaning in life, meaning-making, Long Covid, chronic illness, epistemic injustice

## Abstract

Long Covid (LC) has been called the greatest mass-disabling event in human history. For patients, LC not only has implications for quality of life but also for meaning in life: how one’s life and the world are understood and what is seen as valuable in one’s life. This qualitative empirical study used a Constructivist Grounded Theory approach to investigate the meaning in life of people struggling with LC through ten patient interviews. This study shows that patients lose their prior understanding of life and come to a changed meaning in life, in part due to the experienced (social) isolation and loss of (both physical and cognitive) abilities caused by LC. Moreover, patients struggled with acceptance, uncertainty, and the inherent incomprehensibility and uncontrollability that living with LC entails, though this simultaneously co-existed with hope, optimism and acceptance. Additionally, dimensions of meaning intersect; a patient having some understanding of their illness (dimension of meaning: comprehension) required an understanding Other (dimension of meaning: connection). Emerging from lockdown brought the challenge and isolation of adjusting to chronic illness in society as usual (albeit divided about COVID-19 measures). This study thus offers novel insights regarding changed, present, and sought meaning in life for LC patients.

*“We long-haulers have found ourselves on an involuntary journey that has no map, guidebook, or mileage markers … It is truly a long, lonely trek across the unknown,”* – Miles Griffis, patient.

## Introduction

Considered by TIME magazine “the greatest mass-disabling event in human history” (Ducharme, 2022), Long Covid (LC) poses serious challenges for global and public health (Faghy et al., 2022). LC denotes new, recurring, or ongoing symptoms (Centers for Disease Control and Prevention [CDC], 2021) that occur after a probable or confirmed SARS-CoV-2 infection (Soriano et al., 2022), irrespective of severity of the initial infection (Nurek et al., 2021). Symptoms last for a minimum of two months without alternative diagnosis (Soriano et al., 2022). Symptoms are varied, with respiratory, neuropsychiatric, skeletal muscle and joint, renal, endocrine, dermatological, gastrointestinal, and cardiovascular manifestations (Raman et al., 2022). LC is also defined as a long-term multisystem illness (Nurek et al., 2021).

Across Western Europe, the number of disabled people unable to work as a result of LC has increased dramatically, also leading to substantial economic costs (De Wel, 2022; Kuipers, 2022). Additionally, the pandemic disproportionately affects socioeconomically deprived and marginalized groups (e.g., people of colour, persons with disabilities) and such vulnerable groups also have the highest risk of developing LC (Bambra et al., 2021; Devoto, 2023; Heller et al., 2022; Linehan et al., 2022; Shabnam et al., 2023). Despite this urgency, the widespread impact of LC has so far been overlooked and under-researched. According to Faghy et al. (2022) the restoration of economic and social activities are a driving force behind these decisions. Accordingly, Faghy et al. call for the prioritization of public engagement initiatives which address the increasing burden of LC and to increase the public knowledge and awareness of the remaining threat to global health, in order to avoid the risk of LC becoming an “invisible” or “silent” pandemic (Lavoix, 2021). This includes sustained support for LC research, whereby the novel challenges posed by LC for the lived experience of patients is acknowledged (Faghy et al., 2022).

Interestingly, Callard and Perego (2021) describe LC as the first illness created collectively by an online patient community. Early in the pandemic, patients who suffered from persistent symptoms created the term “Long Covid” (Callard & Perego, 2021). They gathered experiential evidence, advocated for recognition, and initiated medical discussions through collaborative efforts on social media (Callard & Perego, 2021; Roth & Gadebusch-Bondio, 2022). Within months, patients’ individual experiences were transformed into a collective experience recognized as a health problem in public and scientific health discourse (Callard & Perego, 2021; Roth & Gadebusch-Bondio, 2022). Patient-made LC was quickly accepted because scientific knowledge gaps needed rapid filling with experiential evidence and many healthcare professionals are LC patients, amplifying impact and acceptance (Atkinson et al., 2021; Roth & Gadebusch-Bondio, 2022). Despite this initial, essential patient-centeredness and despite burgeoning medical evidence, patients do experience stigma, lack of support, difficulty accessing services and diagnosis, and misunderstanding, dismissal, and disbelief of their symptoms and knowledge (Burton et al., 2022; Callard & Perego, 2021; Ireson et al., 2022; Ladds et al., 2020; McCone, 2022; Rushforth et al., 2021). The dismissal and disbelief can perhaps be attributed to testimonial injustice: the patient is accorded a credibility deficit by the hearer because of a negative identity prejudice held by the hearer (here: a healthcare practitioner with epistemic privilege) (Carel & Kidd, 2014; Fricker, 2011). Additionally, this might have to do with hermeneutical injustice: “a gap in collective interpretive resources puts someone at an unfair disadvantage when it comes to making sense of their social experiences” (Fricker, 2011, p. 1) (here: the lack of understanding and awareness regarding a virus leading to chronic illness and the difficulty of expressing bodily experiences). Narrative inquiry by Rushforth et al. (2021) found that LC patients “struggled with a fragmented inner monologue” (p. 7) due to absent or dismissive professional witnesses to their narrative. One study shows that even doctors with LC struggled to receive care and felt disbelieved and disappointed by their doctors (Taylor et al., 2021). Finding others online with similar experiences lessened patients’ confusion (Burton et al., 2022; Rushforth et al., 2021).

## Meaning in life when living with LC

The present study is a qualitative study of the meaning in life of people suffering from LC. In short, meaning in life can be defined as how one understands one’s life and what is valued as meaningful in one’s life (Brandstätter et al., 2012; Hupkens et al., 2018; Park, 2016). Living with this novel, not yet understood, life-altering chronic illness, in the unique context of a pandemic, with the risk or experience of being disbelieved and dismissed, raises questions about the meaning in life of LC patients. “Meaning in life” is a complex, comprehensive, multidimensional construct (Brandstätter et al., 2012; Derkx et al., 2020; Hupkens et al., 2018). Hupkens et al. (2018) discuss the distinction between “meaning *of* life” and “(personal) meaning *in* life”. Whereas meaning of life denotes cosmic meaning (e.g., religious views), meaning in life denotes experienced personal meaning in one’s life. Brandstätter et al. (2012) offer a systematic review of meaning in life assessment instruments, summarizing definitions of meaning in life as: “a highly individual perception, understanding or belief about one’s own life and activities and the value and importance ascribed to them” (p. 1045). Derkx et al. (2020) build on Baumeister’s (1991) seminal four needs for meaning and propose that meaning in life has seven dimensions: purpose, moral worth, self-worth, control/competence, coherence/comprehension, connectedness, and excitement/wonder/curiosity (see Table I). Park (2010, 2016) describes meaning-making as cognitive and emotional processing to rebuild one’s meaning system: the meaning of a situation is appraised (situational meaning), informed by global meaning (one’s fundamental beliefs about themselves and the world) and how the two align or not, resulting in changes to either the situational or global meaning. This model is used to analyse stressful or traumatic life events such as illness, as illness disrupts one’s global meaning (ordinary way of understanding the world) (Park, 2016).

**Table I.** Dimensions of meaning in life, derived from Baumeister (1991), Derkx et al. (2020), Hupkens et al. (2018), Martela and Steger (2016).

Dimension	Meaning
*Purpose*	Having goals to strive for in life, which one’s current activities can be connected to. Having direction, external goals, inner fulfilment.
*Moral worth, values and justification*	Having underlying values and evaluating one’s actions positively, one’s goals as morally worthwhile.
*Self-worth*	Valuing and accepting oneself and being valued by others for who you are and what you do.
*Control, efficacy, competence*	Feeling competent, feeling influence over one’s life, feeling like one’s choices and actions matter – even if one objectively knows that one has little control over life.
*Coherence, comprehension*	Sensing coherence and order in life, understanding one’s life. Understanding how events and experiences make sense in the larger story of one’s life. This includes a coherent life narrative.
*Connectedness*	Feeling connected to others and/or something other/bigger than oneself. Feeling part of something valuable.
*Excitement, wonder, curiosity*	Feeling excitement, wonder or curiosity. Feeling engaged and motivated rather than purely experiencing the monotony of life.

Frankl (1966), a seminal scholar of the topic, writes of the will to meaning as a natural human force: humans naturally strive for meaning. However, meaning in life is disrupted by stressful or traumatic life events such as (chronic) illness (Bigony & Keitel, 2020; Park, 2010, 2016). Sometimes people are overwhelmingly occupied with surviving, not with meaning-making—as Vosman (2018) critically notes. Frankl (1966) argues that meaning in life supports survival. Thus, it is argued here that survival and meaning-making shift to foreground and background in life with chronic illness. Chronic illness can be seen as a “loss of a life’s map and destination” (Frank, 2013, p. 6), with meaning-making as “a core process for patients adjusting to a chronic illness” (Bigony & Keitel, 2020, p. 107). Those who become chronically ill must adjust: their sense of self, life, narrative, temporality, and beliefs about the world have been disrupted and must be revaluated with cognitive, emotional, and narrative meaning-making processes (Bigony & Keitel, 2020; Bury, 1982, 2001; Carel, 2012; Frank, 2013; Hydén, 1997; Park, 2010, 2016). People are natural storytellers and personal narratives can articulate “the links between body, self and society” (Bury, 2001, p. 281), between past, present, and future—and meaning is derived from this narrative (Bigony & Keitel, 2020; Derkx et al., 2020; Frank, 2013; McAdams, 2001). Narrative meaning reconstruction can occur individually: finding new ways of relating to the body and self and integrating illness into a coherent narrative (Bigony & Keitel, 2020). Reconstruction of meaning can and must also occur socially through co-constructing meaning and narratives with central people in one’s life who hear, believe and understand the narrative, including medical professionals (Bigony & Keitel, 2020). Accordingly, in this study, meaning in life is defined as follows: a multidimensional construct, a highly individual understanding of one’s life (experiences) and world, and a belief with a moral judgement about the value and importance in and of one’s life—attained through intertwined cognitive, emotional and narrative processes (Baumeister, 1991; Bigony & Keitel, 2020; Brandstätter et al., 2012; Derkx et al., 2020; Hupkens et al., 2018; Martela & Steger, 2016; Park, 2010, 2016).

To briefly illustrate, one patient stated: “I am no longer able to be an autonomous person” (Moretti et al., 2022, p. 6), indicating changes in meaning dimensions such as control and self-worth. Patients are still searching for recognition and answers and are trying to accept the loss of their old life (Moretti et al., 2022), thus indicating losses regarding the meaning dimensions coherence and control for example. As patient accounts show: what it means to have LC goes beyond biomedical facts and symptom lists. However, because LC is a relatively new object of study, studies on LC patient experiences are still scant.

All human beings, due to their bodily existence and embedding in the virosphere, are vulnerable and this vulnerability requires a collective, social and political response (Ten Have & Gordijn, 2021). Meaning in life is considered a component of health and overlaps with psychological and subjective well-being (Hupkens et al., 2018). However, a medicalized society dominated by medical expertise, technical sophistication, and science sometimes fails to offer care holistically to its citizens (Bury, 2001). This comes at a cost: patients with their valuable lay knowledge are sometimes dehumanized by medical professionals (Bury, 2001). This entails dehumanization such as objectification and isolation of patients, loss of agency and meaning for patients (Todres et al., 2009). Thus, this study gives indications for supporting meaning in life and humanizing care for LC patients. Furthermore, illness often robs ill persons of their voice and through sharing their story they recover their voice and enable others to speak through their story (Frank, 2013). Illness experiences are difficult to understand and communicate, partially due to a lack of collective hermeneutical resources (Carel & Kidd, 2014; Fricker, 2011). Hermeneutical resources refer to shared tools and social meanings used to interpret social experiences (Fricker, 2011). When hermeneutical resources are lacking, the communicative intelligibility of a speaker is reduced not because of their personal failing but because of an objective difficulty (Fricker, 2011). By creating a space for and analysing LC experiences in relation to meaning in life, this study contributes to collective hermeneutical resources (Fricker, 2011) that render patients’ LC experiences more comprehensible for the storytellers and their listeners.

This study’s aims are therefore twofold, namely 1) to contribute to the accumulation of knowledge of LC experiences and 2) to delineate meaning in life in relation to chronic illness. Accordingly, the main research question of this study is: how do people with LC experience meaning in life?

## Methods

### Research design

Charmaz’s (2014) Constructivist Grounded Theory (CGT) was used as this approach is well suited to studying something as personal and contextual as meaning in life while ill with a novel and uncertain illness. CGT is frequently utilized to study chronic illness (Mohajan & Mohajan, 2022). Charmaz (2006, 2014) describes employing CGT to study varied chronic illnesses—such as heart and circulatory diseases, diabetes, fibromyalgia, and more—in relation to meaning, effects on the self and identity, and time. For example, one study by Najafi Ghezeljeh et al. (2014) employed CGT to study the experience of chronic illness, specifically coronary heart disease. CGT’s ontological underpinning acknowledges reality as constructed by human interaction and interpretation (Charmaz, 2014; Singh & Estefan, 2018). CGT’s epistemological underpinning understands knowledge as socially embedded, as co-constructed by the researcher and participants (Charmaz, 2014; Mills et al., 2006). Thus, CGT does not regard a theory as an objective, external reality discovered in the data by a neutral observer (Charmaz, 2014). Instead, data and theories are shaped and constructed by researchers and participants who are part of and influenced by the world being studied, and theories are an “interpretive portrayal of the studied world” (Charmaz, 2014, p. 17). In CGT, prior works inform and guide—but do not command—the current study (Charmaz, 2014). This study is primarily anchored in social sciences and philosophy literature on meaning in life and chronic illness.

### Research population and sampling

LC is a novel illness about which much is unknown, with many sufferers lacking proof of infection for various reasons (e.g., inaccessible testing). Therefore, this study formulated rather broad inclusion criteria, in accordance with the World Health Organization (2021) clinical case definition: participants had symptoms for a minimum of two months following a probable or confirmed SARS-CoV-2 infection, without alternative diagnosis. People with LC who could share their experiences in English or Dutch were all considered as the research population.

Participant recruitment proceeded through word of mouth and social media advertisements on Instagram and Twitter, inviting long Covid patients to participate in an interview for thesis research. Participants were selected for ease of availability. Additionally, theoretical sampling is essential for CGT and entails “collecting pertinent data to elaborate and refine categories” (Charmaz, 2014, p. 192). Therefore, the researcher included participants for their potential contributions to emerging theory, such as a severely ill patient and a mostly recovered patient. Furthermore, for theoretical and heterogeneous sampling, three men who became ill during the first wave were recruited. Two of them wrote empirical documents in 2020 and these supplemented the interviews. Theoretical sampling was also performed by refining categories by member check. See Table II for participants.

**Table II.** Participants.

Participant	Gender	Age	Occupation	Long Covid	
				*Start of illness*	*Current status*
Anne (NL)	Female	21	Student	January 2022	Mostly housebound*
Bianca (NL)	Female	56	Professor	April 2020	Reduced activities, some recovery
Claire (NL)	Female	20	Nursing student, healthcare worker	November 2021	Mostly recovered
Demi (NL)	Female	20	Student, self-employed	March 2021	Some recovery*
Eric (NL)	Male	23	Student, parttime job	September 2020	Reduced activities, delayed studies, some recovery*
Frances (NL)	Female	21	Student, parttime job	March 2021	Mostly housebound
Gwen (NL)	Female	41	Teacher, sports instructor, consultant	March 2021	Quit teaching job, severely reduced activities as a consultant, on indefinite leave as a sports instructor*
Hugo (USA)	Male	36	Police officer	February 2020	Medically retiring, reduced activities, needs assistance leaving the house*
Miles (USA)	Male	29	Independent journalist	February 2020	Disabled by LC*
Joe (GB-SCT)	Male	36	Self-employed software engineer	March 2020	Significant recovery but continued daily impact*

* *= Participants’ own descriptions*.

### Research ethics

The Ethics Review Committee of the University of Humanistic Studies approved the research plan. According to Dutch law, this study did not require any further ethical review. The study was conducted with continuous regard for the *Netherlands Code of Conduct for Research Integrity* (Universities of the Netherlands VSNU, 2018). The code has “responsibility” as one of its principles. “responsibility” conveys “acknowledging the fact that a researcher does not operate in isolation” (Universities of the Netherlands VSNU, 2018, p. 13) and thus taking into consideration the legitimate interests of participants. Therefore, participants were informed about the study and were asked for their written consent. Moreover, data management was heeded by collecting a minimal amount of data, careful data storage, and using pseudonyms (two participants gave consent to appear by name and approved the use of their citations in the results section). In the case of an interview stirring emotional distress, the researcher had information about available support. The code also contains the principles honesty, scrupulousness, transparency, and independence, which can be summarized as: honest and precise data collection, representation of findings, and reporting on research methods (Universities of the Netherlands VSNU, 2018).

### Data collection

Data was collected through ten semi-structured in-depth interviews of approximately one hour,^1^ conducted in May 2022 by the first author. This allowed the personal experiences of participants to come forward in their words (Creswell, 2013). Meaning in life was explained to and asked of participants as “how you understand your life” and “what is seen as valuable and meaningful in your life”. Moreover, blog posts, opinion articles and essays containing patient reflections were analysed for meaning in life—including empirical documents by doctors with LC and empirical documents by two interview participants. Furthermore, this study included a member check (Patton, 2015). Over email, seven out of ten participants responded to individually check interpretations of their statements and provide further data regarding two emergent findings. Additionally, photo elicitation—an in-depth interviewing tool that stimulates reflections (Patton, 2015)—was used to conclude each interview. Participants were shown seven images and were asked if any of the images represented current/recent meaning in life for them and why. For this, Google Images was used to find stock images that fit the seven dimensions of meaning in life as proposed by Derkx et al. (2020). For example, to represent the first dimension, purpose (having goals, direction), an arrow hitting a target was shown (as this was a stock image result for purpose). To represent the second dimension, moral worth/values/justification, a stick figure holding a scale with a check mark and a scale with an “x” was displayed. To represent the third dimension, self-worth, a person looking in the mirror was displayed.^2^ The aim of showing the images was not meant to validate the seven dimensions but was intended as an elicitation: a prompt for participants to freely associate, to come to further insights regarding their meaning in life.

In CGT, the aim of theoretical sampling is not generalizability or representativeness from sample size, but sampling adequacy (Charmaz, 2014, p. 214). Accordingly, theoretical saturation in CGT is not “witnessing repetition of the same events or stories” (Charmaz, 2014, p. 213). Theoretical saturation can be defined as: “the point at which gathering more data about a theoretical category reveals no new properties nor yields any further theoretical insights about the emerging grounded theory” (Charmaz, 2014, p. 345), one has robust categories and has “defined, checked, and explained relationships between categories and the range of variation within and between … categories” (Charmaz, 2014, p. 213). In line with CGT, we do not claim to have come to a final, objective theory, but a contextual construction based on comparisons between how a few individuals with LC experience meaning in life.

### Data analysis

CGT is largely an inductive approach: theory emerges from empirical qualitative data. However, Charmaz’s (2014) CGT also sees value in inquiry unavoidably being informed by the researcher’s personal and professional insights, existing literature, and sensitizing concepts. These can be used as starting points for data collection and to interrogate and code the data, as long as they do not determine the analysis and emergent theory (Charmaz, 2014). The literature led to the following points of departure for the interview guide and analysis: understanding of life, valued in life, Derkx et al. (2020) seven dimensions, Park’s (2016) global and situational meaning, and Fricker’s (2011) testimonial and hermeneutical injustice.

The interviews were recorded and transcribed by the first author. The transcripts were coded by the first author using ATLAS.ti. In line with CGT, coding of the interviews was done inductively, mostly line-by-line and using gerunds, with an initial and a focused coding phase (Charmaz, 2014). Codes were focused on explicit and implicit meanings, actions, processes, thoughts, and feelings of participants. As Charmaz describes, in CGT she prefers keeping codes “simple, direct, analytic, and emergent” (p. 19) and she does not apply Grounded Theory’s axial coding. Instead, one develops subcategories and shows links between (sub)categories, as one “[learns] about the experiences the categories represent” (Charmaz, 2014, p. 148). She describes: “the subsequent categories, subcategories, and links reflect how I made sense of the data” (Charmaz, 2014, p. 148) and for this she utilizes memo’s. This is the approach we followed. A four-page coding frame is available upon request. The authors discussed interpretations and findings. For each transcript, a memo was written summarizing the findings. 23 memos were written on salient categories and comparisons between data. The memos were then organized to write the findings. Quotes from Dutch interviews were translated.

### Quality and rigour

Due to the explorative nature of this study our focus was on the extent of variation in which the personal experiences occurred, and how exemplary these experiences were, rather than statistical frequency (Corbin & Strauss, 2008). This aim does not mean that our study lacks generalizability, but rather that theoretical generalization, i.e., transference must be separated from statistical significance (Niemeijer, 2015).

So instead of validity, reliability and objectivity, we follow Guba and Lincoln (1989) and Abma (1996) in using six alternative criteria to assess the quality and rigour of our research, namely traceability, recognizability, plausibility, transferability, persuasiveness, and reflexivity. Adopting these criteria automatically implies addressing any potential limitation of our study. CGT is a rigorous methodology, which requires constant methodological reflexivity of all researchers involved. Nevertheless, member checks and purposeful sampling were used for recognizability and plausibility. Memos, audio recording, verbatim transcripts, peer debriefing and staying close to the data help make traceable and reflexive the process of data collection and analysis and the findings (Abma, 1996). The interview guide, use of ATLAS.ti, and consistently applying the analytic (iterative and comparative) methods of CGT also contributed to plausibility (Charmaz, 2014; Thyer, 2001). For transferability, the use of CGT’s inductive line-by-line coding with gerunds forces the researcher to stay open to the world of the participant, rather than imposing prior ideas and forcing the data into pre-existing categories (Charmaz, 2014).

## Results

It seems like [having Long Covid] leads to more meaning-making questions, because life has changed so much. But I find it hard to engage with. Because somehow, I still want things to be the way they were before Covid. And I don’t want to concern myself with new meaning-making questions. (Eric)

As one of the patients (Eric) in this study so poignantly explains in the statement above, LC forces patients to engage with meaning making questions, even if they do not want to.

What follows in this section is the impact of LC on their meaning in life, whereby the main categories that emerged during the analysis will be presented. First, we illustrate the influence of the COVID-19 pandemic as the context of chronic illness onset unique to these patients. Next, changed understanding of life, lost sense of self, the understanding Other, and struggling with acceptance are analysed as aspects of meaning in life. Finally, sources of meaning in life, including hope and optimism, are discussed. Overall, LC led to lost meaning in life, necessitating reflection and meaning making processes to arrive at changed meaning in life.

### The COVID-19 pandemic context

Unique to LC patients is that they became chronically ill during a pandemic. The isolation, uncertainty and controversy of this context influenced their meaning in life by making it harder to understand their illness and leaving them more isolated. There was a sense of “*isolation in isolation*” (Eric): feeling isolated by being ill and lack of understanding and help, while also isolated in lockdown. Experiencing uncertainty was stressed as particularly characteristic of LC: when and where can they get help, why are they so ill, will there be a cure, will it get worse? The pandemic’s strain on healthcare services meant that patients had limited access to healthcare. When they had access, they were often disbelieved by loved ones and medical professionals and/or struggled to receive help. Especially those who became ill in the first wave: they came to doubt their reality, isolated for weeks in case they were contagious, and often finally found answers in online patient communities. Dutch and American patients stated that they had past and recent encounters with unknowledgeable medical staff, even within services dedicated to LC.

[When I found online support groups,] that’s when I started to realize: “oh, this is an illness from Covid that’s not going away and here’s people who also have it.” It’s nice to be able to exchange stories … That made me feel a lot more grounded because I thought that- my doctor at least is sort of telling me “you’re anxious, you’re a hypochondriac”. (Miles)

Furthermore, LC patients became chronically ill from a virus that is familiar to many, leading to others dismissing, misunderstanding, or disbelieving LC as their own COVID-19 experiences were mild and short-lived. As Demi illustrates: “*now you can compare: ‘but I also had [Covid], it was like a flu, a cold. You’re still young … How can you still be so ill from it?’*” Moreover, becoming ill in lockdown meant that patients had yet to experience being ill in society as usual. Once the lockdowns ended, patients felt left behind and struggled to manage their illness. Additionally, patients were confronted by loved ones or people in public spaces denying the existence and/or effects of COVID-19, thus also denying any long-term effects of LC. Patients fear reinfection and/or have come to understand society as ableist and inaccessible.

Now it’s like “good luck, Corona is gone” so there are no possibilities to receive help for Long Covid … I’m only now seeing how I feel within an open society … Things were already difficult while society was halted … And now I have to do all this in a society that’s back to 100% speed. (Eric)

The pandemic context also meant that some patients remain hopeful about the relevance of LC and the momentum bringing forth research and action. Other patients feel dismissed and/or expressed concern about ending up like dismissed patients with other post-viral illnesses.

I think it’s still so underreported and talked about and that’s kind of infuriating, like at least in the States there’s been very little acknowledgement of it from our government … It feels like we’re sort of being erased and being swept under the rug. (Miles)

### Understanding of life

#### Changed understanding of life

LC halted and changed life—challenging patients’ understanding of their life. Patients expressed the following aspects of their understanding prior to becoming ill: one can pursue interests and can influence events in one’s life, being young and/or healthy and thus unlikely to become ill, feeling invincible, and feeling optimistic that (unfortunate) things will turn out alright. As Hugo expressed: “*I thought I was a superhero. I thought I could do anything, and nothing was going to stop me*.” Suffering from LC did not fit with this prior conception. Demi remarked: “*I was taught that you yourself can change things a lot. That cannot be reconciled with running into these obstacles*.” Their entire lives suddenly revolved around illness. Patients thus struggled with the inexplicability and uncontrollability of their illness. The inadequacy of their earlier understanding of life forced them to reflect on meaning in life, which not all patients actively and explicitly did prior to becoming ill.

I think that when I became ill … that’s when you actually start asking questions like you’re asking me about the meaning of life … I had three jobs and I liked them all equally. But suddenly I couldn’t do that anymore. That’s when I realized that work is so important to me … And because I lost that, I noticed: “well, what’s left?” (Bianca)

All this led to an adjusted understanding. This included: acknowledging one’s vulnerability and the fragility of life, seeing the world as unfair, acknowledging one’s (past) privilege, incomprehensibility, and unfortunate things happen and cannot always be controlled.

I thought about how I understand life, and asked: “why me?” … I found it hard to deal with at first … I had bad luck. Anyhow, life is just not fair and these things just happen. I’m accepting that more and more now. That’s easier for me too because I don’t want to be preoccupied with it the whole time. (Anne)

Patients who had been through previous profound challenges seemed to require less adjustment to meaning in life. Furthermore, participants who considered themselves as religious required less adjustment. They expressed finding strength in their belief that God is helping them through and that they can gain something from their challenges. They came to understand illness through their religious worldview. For instance, nursing student Claire emphasized her religious beliefs and stressed learning: “*it has the benefit that I now understand my patients better … For my work it’s only been positive*.” Similarly, Gwen shared:

I don’t believe that it was God’s will that I became ill … but I do believe that once I open myself up to “this happened to me, but it’s a chance to grow … to receive and share love in a different way,” that this can give meaning to the situation. (Gwen)

Non-religious patients also often indicated that they tried to view LC as an opportunity to learn from, derive something positive from, and help other patients. Anne remarked: “*I’m trying to see this as something to learn from and become more grateful*.” However, patients were also cautious of self-deception and denying valid feelings.

There’s a large capacity for self-deception as well, and there’s a strong drive to want to see this experience of illness as something that ultimately serves some deeper purpose and makes me a stronger person. And I think I need to be careful about latching onto that. (Joe)

#### Lost sense of self

Additionally, patients lost some sense of self, as illness obstructed acting upon who they felt themselves to be. This also led to disappointment in oneself, especially regarding achievements and not being there for others. To illustrate, Miles wrote: “*I live a miniature version of my own life and feel like a stump of my former self*,” and this remains true for him today.

#### Understanding and the understanding other

Patients conveyed that their symptoms and experiences are hard to grasp and put into words, with symptom lists never doing the experience complete justice. Patients expressed struggling with understanding the illness themselves, which was compounded by others not understanding their illness. Meaning-making required some understanding of their illness ánd connecting to supportive others who understand them. “Understanding” then means believing, imagining, listening, and supporting. However, for patients, *true* understanding meant having experienced (a similar) chronic illness. As Miles remarked: “*Even though you can have support from friends, family, or partners, it’s still very much like no one’s going to have your exact symptoms or experience with chronic illness, so I do always feel somewhat isolated*.” Misunderstanding or dismissing the impact of LC was most painful when it came from loved ones who had seen the impact up close. Occasionally this led to avoiding or breaking off contact. Furthermore, patients spoke of being disbelieved or dismissed by doctors, although some did have supportive doctors. Certain patients remarked that they avoided speaking about LC: it either felt too complex to explain and/or incomprehensible to others, or seemed forgotten by society, or seemed to make people without LC uncomfortable. Occasionally, certain people (including doctors) would frame their symptoms as burnout, anxiety, or lack of effort. Patients also feared and experienced being viewed as difficult, complaining, contriving, or seeking attention.

I always feel like the person sitting opposite me thinks that I’m telling a bullshit story … because it’s so unclear … I try to think about what I feel but I can’t yet and there aren’t words for it yet because it’s just so new. So it’s very frustrating because I’d like to have a picture and say “this is what’s going on with me” but I don’t think that will come. (Eric)

For some, contacting fellow patients was valuable: it helped them understand their own experience and feel less lonely, and it gave them purpose in the sense of helping other patients. Other participants struggled with contacting fellow patients: experiences did not resonate with their own experience, or it lead to unhelpful comparison with people who are better or worse off, or it did not offer definitive answers, or it was simply (too) upsetting to read about suffering.

In the first year of being severely affected … I was using these groups for longer than I thought was healthy … I was using it as a kind of emotional balm … It can be very easily addictive. Because it doesn’t get you the thing that you really need, which is an answer. (Joe)

#### Struggling with acceptance

Difficulties with acceptance due to the illness being so unpredictable, uncertain and incomprehensible were often mentioned. Patients spoke about accepting that things are more difficult and take time, while also expressing that it is easier said than done. They spoke of becoming more grateful, but also feeling frustrated with having so little. Patients mentioned blaming themselves for not trying hard enough and then pushing themselves, resulting in confrontational symptom flare-ups: “*You can’t push through. Your body is not letting you*” (Hugo). For some, working on acceptance is the meaning made from being ill. For example, Bianca expressed: “*Actually, it’s all a big lesson in becoming Zen. By which I mean that you can kind of accept what’s going on*.” It remains difficult to find meaning in an experience of suffering and loss so hard to accept.

Every time it’s that fight with acceptance. And I still notice sometimes that I’m frustrated and I think “why is everything so difficult?” Especially if I consider how I lived before this. It’s such a big contrast, it’s almost unfathomable. (Gwen)

### Sources of meaning in life

Loss and necessitated reflection due to illness also led to reappraising what they valued as meaningful in life. Often, patients’ sources of meaning remained but the way they manifested changed. To illustrate: “*[Achievement and proving myself] are still important. But now I need to reshape this because I’ve noticed that how I used to do it … it’s untenable now*” (Demi). Sometimes patients expressed, as Bianca put it: “*there’s an old repertoire of meaningful things … And there’s a new repertoire of meaningful things*.” Frequently, this meant valuing work and performance less and valuing self-care and relationships more. Additionally, past health, past or current privileges, and things taken for granted became more valued. Changes in sources of meaning in life were often not by choice, but by necessity from lacking abilities, support, and answers. Additionally, sometimes patients lacked the energy and cognitive functioning to reflect on and seek meaning in life. Loss of meaning was also expressed, such as by Frances, tearfully: “*At a certain point, when you’ve had so much pain for so long, all of that [which I cared about] doesn’t matter at all anymore*.” Patients conveyed that incomprehensibility and lack of control made their efforts feel pointless at times.

Certain patients foresee a different future. For example, Frances cried as she spoke about giving up her goal of becoming a teacher. Should they recover, Bianca and Gwen intend to work fewer hours and value work less. Some find the future too uncertain, confronting the question: “what if I do not get better?” Additionally, lacking knowledge about their illness’s cause, trajectory, and unpredictable and debilitating symptoms makes it difficult to work on bigger goals and look long-term. Instead, patients are forced to view life more day-by-day, managing symptoms and working with changing limits each day. Demi mentioned, for instance: “*my meaning in life used to be focused more on the future, but now it is more focused on the present*.” Looking ahead is simultaneously helpful and disappointing.

It’s harder to look ahead now. I really want to be able to think about when I’m doing better but every time … it’s extra hard because you were set on that it would work out but you’re still sick. It’s with mixed feelings to look ahead. (Anne)

Experienced, changed, and lost sources of meaning that were mentioned can be seen in Table III.

**Table III.** Sources of meaning.

Connections	Interests, activities, joy	Illness-related	Influence and orientation
Relationships with supportive loved ones	(Small) joys, improvements, accomplishments	Advocating	Independence and freedom
Giving love	Hobbies	Researching illness	Control
Helping others	Work	Knowledge/answers and comprehension regarding illness	Setting goals and making plans
Contacting fellow patients	Studies and learning about areas of interest	Seeking treatment	Purpose
Connecting with natural surroundings	Passion for projects	Learning from the illness experience	Focusing on the present
Sense of self	Philosophy	Gaining something positive from the negative experience	Hope
Religion		Contacting fellow patients	Optimism

To illustrate some dimensions of meaning in life, Miles spoke of his purpose as raising LC awareness and reading research: “*At least I’m able to try to fight this in some ways rather than just sit back and watch. It gives me a little bit of purpose in some ways*.” Moreover, often sources of meaning overlapped or intersected. Such as in this example that illustrates control, comprehension, and setting goals in response to the question what is difficult about meaning in life:

Things happen to you and … you have to deal with that, and you can’t really do anything about it. It makes me think: “I really wanted this … but it’s not possible and I can’t give a good reason.” Sometimes it contains some frustration, I miss that I can’t always set the goals for myself that I’d like to set … You want to blame something. Sometimes I blame myself because I think “maybe I should just push a little harder.” But then I notice that my body says no. (Demi)

#### Hope and optimism

Furthermore, patients described experiencing or seeking meaning through hope and optimism: their health will improve, next month could be different, research will offer answers, they will emerge from this having learned something. For instance, one affected doctor reflects: “*As I continue to live with uncertainty, fear … the silver lining is the hope that my future patient interactions will allow me to provide greater comfort and validation*” (Siegelman, 2020). Eric, when asked during photo elicitation which image currently represents meaning in life for him, replied:

[Image 7] … Dreaming about nice things. Being happy. So really keeping the optimism and thinking “it will be okay” … Making plans whether they happen or not but at least trying … Regarding meaning in life, this is what I’d want to be more and what I try to do more.

Patients expressed that initially they were hopeful they would soon recover. As time passed, hope became more difficult, there was disappointment, and working on acceptance followed.

I’ve always been very hopeful … I’ve always had the help of the expectation that this would turn out alright. But after 14 months of struggling … I’m starting to think “will it ever be alright?” And I’m processing in my head: how bad would it be if it doesn’t turn out alright? (Gwen)

## Discussion

### Sources and intersections of meaning in life

This study provided answers regarding changed, present, and sought meaning in life for people with LC. Sources of meaning were also identified. These findings can be viewed through the lens of the dimensions of meaning in life (see Table I) discussed in the Introduction. See Table IV for an illustration of how the findings align with the dimensions of meaning in life.

**Table IV.** Findings compared with dimensions of meaning in life (Derkx et al., 2020).

Purpose	Moral worth	Self-worth	Control
Patients mentioned advocating and/or helping others as their purpose.	Patients saw loving and caring for others as the right and most important thing to do (even though this is difficult due to illness or hard to accept when all else is lost).	Patients struggled with not being able to perform and achieve and be there for others.	Patients struggled to understand life while lacking control over their illness.
Coherence/ comprehension	Connectedness	Excitement/wonder/curiosity	
Patients sought an explanation for their illness (situational meaning derived from global meaning) or had to adjust their understanding of life (global meaning) (Park, 2016).	Patients needed the support and understanding of others and patients find good relationships more important than ever.	Patients held onto their passions, interests, and (small) joys.	

Furthermore, what emerged in this study is the importance of these sources/dimensions of meaning intersecting. Sometimes it was not a matter of a patient seeking both comprehension and connection (comprehension + connection). The patient sought the exact intersection of these dimensions: their own comprehension depends on the illness being somewhat comprehensible for supportive others (comprehension x connection). Something similar was described by Rushforth et al. (2021): patients’ fragmented inner monologue due to lack of an empathetic audience. Derkx et al. (2020) also observed that experiencing other dimensions seemed dependent on connectedness, recommending further research. This points to how humans are relational and interdependent (Tronto, 1998, 2014). Similarly, here meaning in life was defined as involving narrative processes: people as storytellers make meaning (of illness) through narratives with both individual and social processes (Bigony & Keitel, 2020; Bury, 1982, 2001; Frank, 2013; Hydén, 1997; Rushforth et al., 2021). In the interviews, meaning-making and meaning-seeking were seen in action as patients shared their disrupted narratives and described struggling to co-construct the narrative with others. Additionally, the interview questions seemed helpful for constructing the narrative and meaning-making: patients sometimes expressed that they had not yet considered such a question, before coming to an insight.

Moreover, hope and optimism—believing that things will turn out alright and/or something good will come of the experience—seemed important meaning sources. This partially overlaps with the dimension “purpose”, which is often defined rather goal-oriented (striving for goals, inner fulfilment) but also (more similarly to hope) as “[connecting] current activities to a valued future state or aspired perspective” (Derkx et al., 2020, p. 40). Hupkens et al. (2018) regard optimism as significant for meaning-making, it is “a basis for meaning in the future” (p. 8). Research shows that meaning in life predicts or enhances hope and optimism (Mascaro & Rosen, 2005, 2006; Wong, 2012), while it is also argued that hope (of reaching goals) is necessary to experience meaning in life (Feldman & Snyder, 2005). However, as Beeris et al. (2022) point to in their research on resilience, there is a danger that a too narrow understanding of constructs such as resilience and hope and the role they play in meaning in life can unintentionally forge a dichotomous narrative of “a meaningful future,” that is, one needs to live life in a “hopeful way” in order for it to be meaningful.

Furthermore, acceptance seems operative in meaning in life. Acceptance relates to control and comprehension: patients struggled with accepting a lack of control and comprehension. Additionally, patients comprehended illness as an opportunity to develop acceptance: acceptance as the meaning made. Park (2010) also reports that acceptance is a common meaning made. Similarly, Baumeister (1991) and Derkx et al. (2020) discuss “interpretive control”: control exercised through how the situation is understood (rather than truly changing the situation), which makes acceptance easier. Accepting lack of control could be an attempt at regaining some control and thus meaning. However, surely not all of acceptance’s role in meaning in life can be equated to control and comprehension. Perhaps, moments of acceptance of something that is not entirely under control and comprehended are possible.

### Epistemic injustice

Unfortunately, the dismissal, disbelief, and lack of help reported in studies (Ladds et al., 2020; Rushforth et al., 2021) persist today. Since already marginalized and disadvantaged groups are most likely to develop LC (Devoto, 2023; Shabnam et al., 2023), the risk of LC patients experiencing epistemic injustice is even more pressing. Our findings indicate that testimonial injustice (Carel & Kidd, 2014; Fricker, 2011) was experienced by patients. Patients were deemed as uncredible knowledge sources, their testimony of chronic illness due to coronavirus dismissed as hypochondria, for example. Hermeneutical injustice (Carel & Kidd, 2014; Fricker, 2011) was also seen. Take Eric, who felt disbelieved and expressed: “*I try to think about what I feel but … there aren’t words for it yet*.” First, society and medical practice does not always give credence to what cannot be fitted into existing models of understanding. Second, it is hard to render chronic illness, fatigue, and pain comprehensible for someone who is not ill—whether they are willing to understand, or they are unwilling to understand out of disbelief and discomfort. Both can be related to Wendell’s (2013) myth of control: people desire control and many are therefore unwilling to believe that people, despite all personal and medical efforts, can remain ill from a virus. Instead, Kielanowski argued for “the right to be ill”: against excluding the ill, blaming the ill for their illness and framing health as a moral imperative (Halasz, 2018). Additionally, Frank (2013) argues that narratives about illness and the body are hard to tell: “the body eludes language” (p. 2)—the body speaks in pains and symptoms, but we cannot articulate this. Not being recognized as a credible knowledge source and/or not being able to render experiences comprehensible causes isolation from others (impacting connectedness), incomprehensibility (impacting comprehension), self-doubt (impacting self-worth), and lack of control and ability to pursue goals (impacting control, purpose). Meaning was also made of experienced epistemic injustice: some participants now advocate for patients, from which they derive purpose for example. Finally, as noted in the introduction, LC must be considered beyond biomedical facts and symptom lists, as meaning in life is a component of health worthy of attention for more holistic, humanizing care that upholds connection, agency and meaning for patients. As mentioned, this requires the ability to co-construct meaning and narratives with people in one’s life, which in turn requires what Fricker (2011) calls collective hermeneutical resources to render illness experiences more comprehensible. This would also be in line with person-centred care, which “captures the person’s suffering in an everyday context, in contrast to medical narratives that reflect the process of diagnosing and treating the disease” (Ekman et al., 2011, p. 250).

### Limitations and strengths

CGT does not aim to discover *the* objective reality, the aim is to discover *a* co-constructed reality (Charmaz, 2014). It is thus ill-suited to evaluate qualitative research such as CGT according to external validity (i.e., statistical frequency, acontextual generalizability) because the research seeks contextual understanding (Charmaz, 2014; Niemeijer, 2015; Smith, 2017). The value of this study instead lies in petite generalizations (themes and patterns within a particular case/context) (Stake, 1995) that contribute to theory on meaning in life with chronic illness; and in transferability (direct testimony, rich descriptions and interpretations leading to readers considering contextualized findings relevant for their context) (Smith, 2017; Tracy, 2010). Contextualized generalizations were made to answer how meaning in life is experienced by people with a novel illness in a unique pandemic context and to offer hermeneutical resources to render patients’ LC experiences more comprehensible. Finally, theoretical sampling was limited in a study of this scope and duration. Findings could have been further refined in a more expansive study.

### Recommendations

Further expansive longitudinal qualitative research, which takes the complexities of living with a post-viral disease such as LC fully into account, is imperative. Ideally, such research would facilitate patients in fostering understanding together regarding what meaning in life with this (and other) post-viral illness(es) means. In this study, patients were more reliant upon themselves as the researcher limited influencing participants with theory. Considering our findings regarding co-constructing the narrative with understanding others, further research could include interviewing loved ones and caregivers of people with LC. Moreover, further research could focus on the intersection of dimensions, similar to how Derkx et al. (2020) recommend investigating the overlap and intertwinement of dimensions, including the constitutive function of connectedness and the relations between acceptance, hope, optimism and meaning in life. Patient care also involves addressing the need for meaning in life which is affected by LC. The findings give indications for how patients can be offered more humanizing care. When disease and disability are merely seen as failures of medicine, medicine is reduced to a health technology and dehumanizes the patient (Halasz, 2018). Patients’ LC experiences must be acknowledged in their totality to support meaning in life and humanization of patients.

## Conclusion

To our knowledge, this study is the first to explore how people with LC experience meaning in life. As our findings show, meaning in life changed substantially for people with LC since becoming ill. Losing abilities and set givens, led patients to have a different understanding of their life and world. What is more, the COVID-19 pandemic complicated meaning in life due to (social) isolation and (constant) uncertainty. Patients felt isolated due to illness, while doctors were inaccessible and/or dismissive or unable to explain the illness, and others disbelieved or misunderstood LC—all while isolated in lockdown in a changed world. The end of lockdown once more challenged meaning in life: struggling and feeling left behind as society continues as usual. Having LC also led to new awareness of injustice, inaccessibility, and ableism in society and feeling a need amongst patients to advocate for them. All this necessitated reflection on and changed meaning in life—although this renewed meaning and different life remain hard to accept and understand. Nevertheless, LC was also often understood as an opportunity or challenge to learn from and gain something from: realizing what is important, learning to deal with hardships, learning to put oneself first, acceptance, and gratitude. For people with this relatively novel illness, uncertainty, acceptance and hope thus seem to alternate and co-exist in sought for and present meaning in life.

## Supplementary Material

LN 2023 Interview guide Supplementary.docx

## Data Availability

The authors confirm that the data supporting the findings of this study are available within the article according to FAIR principles (Findability, Accessibility, Interoperability, and Reusability).

## Notes

1. One of the ten participants was too ill for an interview and thus answered the interview guide questions in voice memos totalling over an hour, with follow-up questions answered in writing.

2. The full selection of images can be provided by the authors on demand.
