# Supplementary material for: Lost and changed meaning in life of people with Long Covid: a qualitative study
Source: Int J Qual Stud Health Well-being. 2023 Dec 6;19(1):2289668. doi: 10.1080/17482631.2023.2289668 (PMC11737832; doi:10.1080/17482631.2023.2289668)
Supplement: LN 2023 Interview guide Supplementary.docx [file ZQHW_A_2289668_SM7782.docx]

## Interview guide

**1a.** If a good friend of yours were to describe you, how would they describe you?
(Opt.: **1b.** Can you tell me what it’s been like for you, being ill with Long Covid?)

*This study is about meaning in life for people with Long Covid. In short, meaning in life and meaning-making have to do with: 1) how someone understands their own life 2) what is valued as meaningful, what is important/valuable in one’s life.*

**Before Long Covid**

**2.** How did you understand (or not understand) your life? How did you understand what happened in your life, or why you did the things you did? What did you see as given, obvious, self-evident about your life?

**3.** What made your life meaningful? What did you value/what was important in your life?

**Since Long Covid**

**4.** How do you (or do you not) understand your life since becoming ill with Long Covid? How do you explain to yourself and to others what has happened and what you are now dealing with?

**5.** What is meaningful in your life since you’ve become ill with Long Covid?

**Other questions**

**6.** Unfortunately, a lot is still unknown about Long Covid. Additionally, you became ill in a unique, turbulent, uncertain context: the pandemic. Has this pandemic context influenced how you experienced being ill? If so, how?

**7.** Have you experienced talking to friends, colleagues, family, partner, doctors about Long Covid, and you did not feel heard, believed, or understood? If so, can you tell me about that?

**8.** Have you experienced difficulties in expressing to others what you now deal with, and/or that it is difficult for others to understand what you experience? If so, can you tell me about that?

**9. Images:** Please select an image, if any, that represents “meaning in life” for you currently. Can you explain why you chose it? / Do any of these images remind you of a recent situation in which you experienced “meaning in life”?

**10.** Is there anything left that you would like to say or ask?
